# Exosomes in Cancer: The Biology, Function, Diagnosis, and Therapy

**DOI:** 10.1002/mco2.71074

**Published:** 2026-10-21

**Authors:** Jun Wu, Lei Wu, Kang Yang, Kang Xie, Tao Lu, Tiangxiang Huang, Dengfeng Wan, Tao Song, Longbo Zhang

**Affiliations:** ^1^ Departments of Neurosurgery National Clinical Research Center for Geriatric Disorders (Xiangya Hospital) Central South University Changsha China; ^2^ Department of Neurosurgery The Second Affiliated Hospital of Nanchang University Nanchang China; ^3^ Departments of Neurosurgery National Regional Center for Neurological Diseases Xiangya Hospital Central South University Jiangxi China

**Keywords:** biomimetic nanovesicles, exosomes, extracellular nanoparticles, multifluid liquid biopsy, pan‐cancer hallmarks, precision oncology, therapeutic interception

## Abstract

Exosomes are central mediators of intercellular communication in the tumor microenvironment, transferring proteins, lipids, metabolites, and nucleic acids that regulate cancer progression. Recent advances in multi‐omics and extracellular vesicle profiling have revealed substantial molecular and functional heterogeneity, extending beyond classical membrane‐bound exosomes to nonmembranous extracellular nanoparticles such as exomeres and supermeres. Here, we provide a pan‐cancer synthesis of exosome biology, integrating mechanisms of biogenesis, cargo sorting, and secretion with emerging concepts of extracellular nanoparticle diversity. We examine how tumor‐derived exosomes remodel local and distant microenvironments by promoting angiogenesis, metabolic reprogramming, immune suppression, premetastatic niche formation, and cancer‐associated cachexia. We also discuss the relative contributions of active organotropic targeting and passive spatiotemporal accumulation to metastatic dissemination, together with tissue‐specific mechanisms governing endothelial barrier crossing, myeloid‐cell reprogramming, and intercellular network integration. Clinically, we evaluate exosome‐based liquid biopsy, molecular subtyping, engineered delivery systems, and strategies that inhibit endogenous vesicle production or circulation. Finally, we outline key translational challenges, including single‐vesicle heterogeneity, limited intravital tracking, and the absence of standardized cGMP manufacturing and analytical frameworks across diverse tumor types and clinically relevant biological contexts worldwide. This review defines a roadmap for exosome‐guided precision oncology.

## Graphical abstract

Tumor‐derived exosomes and nonmembranous extracellular nanoparticles (exomeres/supermeres) constitute a heterogeneous communication network that drives cancer progression. By shuttling functional biomolecules across tissue‐specific barriers, these nanoparticles orchestrate local microenvironmental remodeling, immune suppression, premetastatic niche formation, and systemic cachexia. Clinically, this network offers dual utility: single‐vesicle liquid biopsy for longitudinal tracking, and engineered/intercepted vesicles as novel targets for precision oncology.

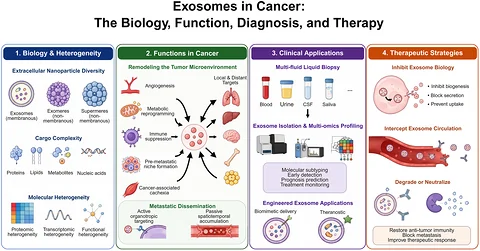

## 1 Introduction

The orchestration of neoplastic progression extends far beyond the cell‐intrinsic mutations of malignant clones, fundamentally relying on a dynamic and reciprocal communication network within the tumor microenvironment (TME) [1]. Over the past two decades, extracellular vesicles (EVs), particularly exosomes, nano‐sized membrane vesicles (40–150 nm) of endocytic origin, have emerged as pivotal mediators of this horizontal intercellular communication [2]. Shuttling a complex cargo of proteins, lipids, metabolites, and diverse noncoding RNA species, exosomes are actively secreted by tumor cells and stromal constituents alike [3]. Upon internalization by recipient cells, these bioactive vehicles effectively reprogram local and systemic landscapes, driving classical hallmarks of cancer such as sustained proliferative signaling, metabolic rewiring, angiogenic sprouting, and immune evasion [4, 5]. Importantly, recent multi‐omic and high‐resolution separation breakthroughs have shattered the archaic view of exosomes as a homogeneous populace, revealing a highly sophisticated spectrum of structural and size heterogeneities that spans from classical membranous vesicles to newly identified, nonmembranous extracellular nanoparticles (ENPs) including exomeres and supermeres [6]. This structural diversity establishes the tumor secretome not merely as a compilation of passive metabolic byproducts, but as a diverse armada of circulating metabolic catalysts that dictate the macroevolution of pan‐cancer landscapes [7].

However, as these heterogeneous vesicular populations diffuse from the primary nidus, their functional deployment is strictly governed by the physiological realities, anatomical matrices, and spatial heterogeneities of the host stroma. This geographic determinism establishes a multidimensional matrix of tissue‐specific complexities across the pan‐cancer spectrum [8]. Rather than operating in a uniform physiological vacuum, tumor‐derived exosomes must navigate distinct regional constraints, ranging from the highly vascularized, compliant stromal compartments of peripheral epithelial tissues to the uniquely insulated, dense macromolecular matrices protected by specialized neurovascular units and tissue‐specific immunological sentinels. This spatial heterogeneity forces a critical scientific debate within the field of cancer nanomedicine: whether remote niche preparation and barrier crossing are driven by active, ligand‐mediated organotropic homing, or accelerated by passive spatiotemporal leakage and hemodynamic entrapment within compromised vascular beds [9, 10]. Dissecting these diverse tissue‐specific mechanics is fundamentally critical; it explains how a single malignant lineage or localized clone customizes its extracellular vesicle molecular artilleries to compromise varied physiological barriers and subvert local homeostatic cell types across the entire human body.

An urgent, high‐level synthesis is therefore required to bridge general pan‐cancer exosomal principles with these distinct spatial and structural complexities. While exceptional reviews have individually detailed the biochemical cargo loading of systemic vesicles or the narrow clinical application of specific tumor isolates, there remains a critical knowledge gap in systematically integrating how these conserved extracellular pathways are uniquely modified, refined, and deployed when confronting the most formidable anatomical and immunological constraints of the host organism [11]. Investigating these cross‐cancer commonalities alongside extreme tissue‐specific heterogeneities is not merely an academic exercise; it holds the key to uncovering universal diagnostic liquid biopsy biomarkers and engineering next‐generation, target‐specific biomimetic vehicles capable of traversing strict biological barriers for precision drug delivery [12, 13].

This review provides a comprehensive, pan‐cancer synthesis of the biology, function, diagnosis, and therapy of exosomes, harmonizing macrolevel universal mechanisms with high‐resolution spatial and structural complexities. We begin by delivering a consolidated inventory of the diverse molecular cargo species encapsulated within cancer exosomes, expanding into the newly discovered realms of exomeric and supermeric structural heterogeneities and their selective intracellular packaging. We then dissect the canonical roles of these vesicles in driving TME remodeling, angiogenesis, and metabolic reprogramming across major solid and hematological malignancies, including a dedicated evaluation of the active versus passive organotropic homing debate and systemic host wasting syndromes like cachexia. These general principles are subsequently examined through the lens of maximum tissue‐specific complexity, exploring how exosomal networks are uniquely deployed to cross tight vascular barriers, reprogram tissue‐resident immune cells, and integrate into functional host cellular circuitry. Finally, we review the global translation frontier, evaluating exosome‐based liquid biopsy platforms across multiple biofluids, advanced therapeutic delivery vehicles bridging natural vesicles with synthetic hybrid liposomes, and innovative defensive strategies aimed at pharmacological biogenesis inhibition and extracorporeal circulatory interception. Through this unified multidimensional framework, we aim to offer a definitive blueprint that guides the future of exosome‐targeted precision medicine.

## 2 Biogenesis, Secretion, and Molecular Cargo Inventory of Cancer Exosomes

### 2.1 The Canonical Cascades of Exosome Biogenesis and Secretion

The intracellular journey of exosomes is a highly orchestrated cytological process that distinguishes them from other extracellular vesicles, such as microvesicles formed by direct plasma membrane budding or apoptotic bodies resulting from programmed cell death. Exosome biogenesis initiates within the endosomal system through the inward invagination of the clathrin‐coated plasma membrane, leading to the formation of early endosomes. As these endosomes mature into late endosomes, their limiting membranes undergo secondary inward budding, encapsulating specific cytosolic components, proteins, and nucleic acids into intraluminal vesicles (ILVs). This morphological transformation converts the late endosome into a multivesicular body (MVB) [14, 15].

The sorting of molecular cargo into ILVs and the concomitant driving of membrane invagination are governed by two distinct yet cooperative mechanisms: the Endosomal Sorting Complex Required for Transport (ESCRT)‐dependent pathway and the ESCRT‐independent pathway [16, 17]. The canonical ESCRT machinery comprises four multiprotein complexes (ESCRT‐0, ‐I, ‐II, and ‐III) acting in a chronological cascade alongside associated ATPases such as VPS4. ESCRT‐0 recognizes and clusters ubiquitinated intracellular proteins on the endosomal membrane, subsequently recruiting ESCRT‐I and ‐II to initiate membrane deformation into a bud. Finally, ESCRT‐III drives the constriction and scission of the vesicle neck, sealing the newly formed ILV inside the MVB [18]. Concurrently, tumor cells frequently exploit ESCRT‐associated accessory proteins, such as ALIX (programmed cell death 6 interacting protein) and syntenin‐1, to fast‐track the sorting of oncogenic receptors (e.g., syndecan, EGFR) directly into developing ILVs, promoting a highly malignant vesicular phenotype [19]. Conversely, the ESCRT‐independent pathway relies heavily on specialized lipid microdomains known as lipid rafts, which are highly enriched in sphingolipids and cholesterol. Within these domains, neutral sphingomyelinase 2 (nSMase2) hydrolyzes sphingomyelin into ceramide [20]. The cone‐shaped geometry of ceramide spontaneously induces inward curvature of the endosomal membrane, facilitating ILV budding without requiring the bulky ESCRT protein machinery. Furthermore, tetraspanins, a family of four‐transmembrane domain proteins including CD63, CD81, and CD9, are ubiquitous orchestrators in this independent pathway. Tetraspanins form dynamic web‐like networks (tetraspanin‐enriched microdomains, TEMs) that cluster specific receptors, adhesion molecules, and cytosolic RNAs, effectively directing their sorting and packing into the invaginating ILVs [21, 22].

Following MVB maturation, the intracellular fate of these structures bifurcates: they are either directed to lysosomes for degradation and recycling or trafficked to the plasma membrane for exocytic release (Figure 1). In malignant cells, the balance is heavily tilted toward exocytosis to fuel autocrine and paracrine signaling loops. This trafficking is directed by the Rab family of small GTPases. Specifically, Rab27a and Rab27b facilitate the transport and docking of MVBs along the cortical actin cytoskeleton toward the plasma membrane, whereas Rab11 and Rab35 control distinct recycling or specialized secretion routes [23, 24]. Upon docking, the final fusion of the MVB limiting membrane with the plasma membrane is executed by specific soluble N‐ethylmaleimide‐sensitive factor attachment protein receptor (SNARE) complexes (e.g., SNAP‐23, VAMP‐7, and Syntaxin‐4) [25]. This fusion event abruptly releases the internal ILVs into the extracellular space, where they are officially designated as exosomes.

**FIGURE 1:**
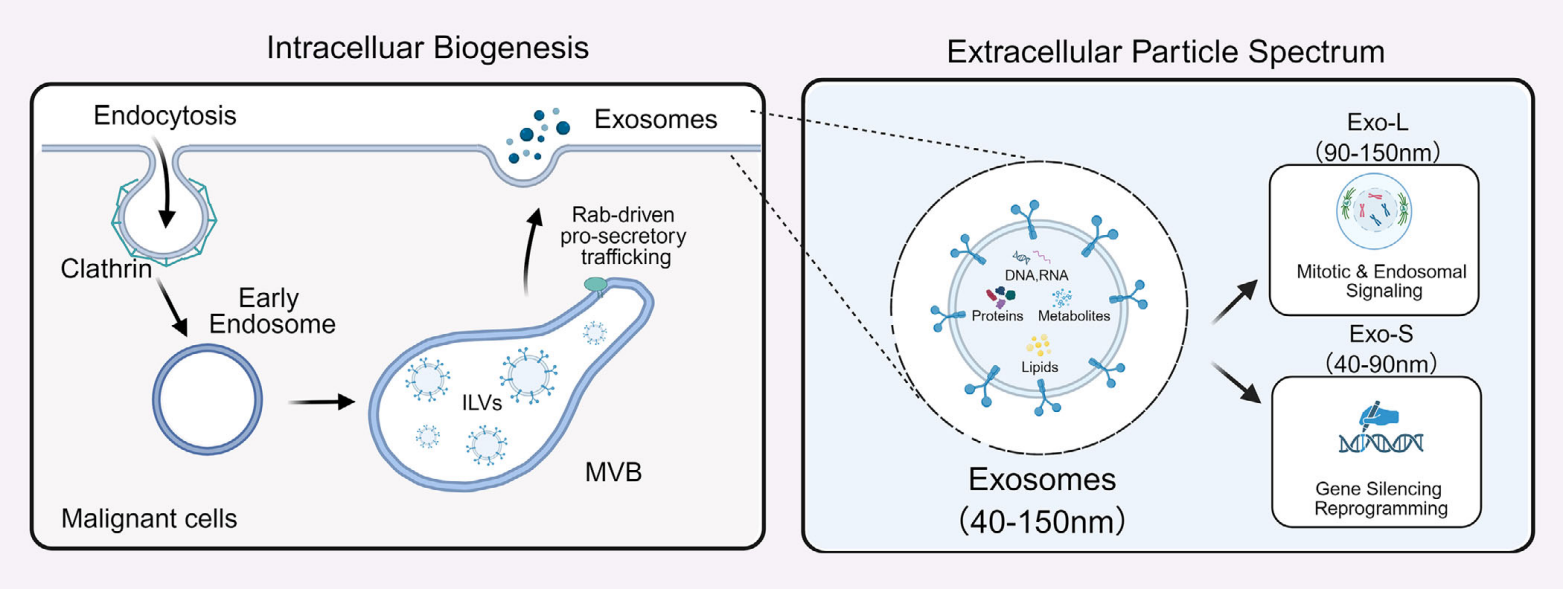
Biogenesis, secretion, and extracellular heterogeneity of cancer‐derived exosomes. Exosomes originate through clathrin‐mediated endocytosis, early endosome formation, and inward budding of the endosomal membrane to generate intraluminal vesicles (ILVs) within multivesicular bodies (MVBs). Rab‐dependent pro‐secretory trafficking directs MVBs to the plasma membrane, where fusion releases ILVs as extracellular exosomes. In the tumor secretome, exosomes span a heterogeneous size spectrum of approximately 40–150 nm, including small exosomes (Exo‐S, 40–90 nm) and large exosomes (Exo‐L, 90–150 nm), which carry distinct molecular cargos and mediate gene‐regulatory, mitotic, endosomal, and intercellular signaling programs. *Source*: Created with BioRender.com.

### 2.2 The Molecular Cargo Landscape

For over a decade, canonical literature conceptualized the lumen and lipid bilayer of cancer‐derived exosomes as high‐density repositories for active oncogenic proteins, RNAs, and metabolic enzymes. However, a revolutionary paradigm shift, spearheaded by landmark high‐resolution proteomic and transcriptomic reassessments [26], has vigorously challenged these historical assumptions. This rigorous metrological re‐evaluation demonstrated that many functional biomolecules previously attributed to classical endocytic exosomes are, in reality, components of distinct, nonmembranous ENPs that co‐purify during standard bulk isolation (Figure 1) [27]. Therefore, a modern pan‐cancer synthesis requires an accurate segregation of what is genuinely encapsulated inside the exosomal bilayer versus what is bound to peripheral nonmembranous carriers.

#### 2.2.1 The Extracellular RNA (exRNA) Profile

The precise molecular cartography of extracellular RNA (exRNA) species within the tumor secretome remains one of the most intensely debated arenas in vesicle biology. While it is undeniable that tumor cells utilize circulating RNAs to horizontally manipulate peripheral stromal and parenchymal niches, classical assertions that small noncoding RNAs, particularly microRNAs (miRNAs), are universally concentrated and encapsulated within endocytic exosomes have faced rigorous metrological skepticism [28]. A foundational paradigm shift in the field was established by high‐resolution density gradient fractionation and direct immunoaffinity capture analyses, which forced a comprehensive reassessment of exosome composition. This landmark study demonstrated that the absolute molecular constituents of extracellular matter are strictly compartmentalized between true, membrane‐bound exosomes and nonvesicular extracellular compartments:

##### 2.2.1.1 The Nonvesicular and Exomeric Segregation (Small exRNAs and Processing Machinery)

Crucially, the fundamental small RNA processing machinery, specifically Argonaute proteins 1 to 4 (Ago1–4), along with standard canonical glycolytic enzymes (e.g., GAPDH) and cytoskeletal proteins, was not detected in true immuno‐isolated exosomes [29]. Because Ago proteins serve as the mandatory chaperones for functional miRNAs, their exclusion from the exosomal lumen indicates that the vast majority of circulating small noncoding RNA species, including historically characterized oncogenic vectors like miR‐21 or targeted therapy resistance mediators like miR‐221/222, are primarily stabilized and transported outside the classical lipid bilayer [30, 31]. Instead, they exist within nonvesicular macromolecular complexes or nonmembranous nanoparticles (such as exomeres and supermeres). When studying spatial heterogeneities or systemic hallmarks (such as the localized transfer of miRNA‐1246 or miR‐10b in specialized high‐grade microenvironments), researchers must consider that these rapid, phenotypic alterations may be driven by dense nonvesicular protein–RNA aggregates rather than intra‐vesicular transport [32, 33].

##### 2.2.1.2 The Genuine Intraexosomal Fraction (Differential Long exRNA Expression)

Conversely, RNA‐Seq profiling across high‐resolution fractions confirms that extracellular RNA is differentially expressed between pure exosomes and nonvesicle domains. Membrane‐enclosed exosomes preferentially prioritize and preserve high‐molecular‐weight transcripts, including fragmented structural RNAs, specific long noncoding RNAs (lncRNAs), and circular RNAs (circRNAs) [34, 35]. The selective packaging of these long transcripts is actively facilitated by specialized RNA‐binding proteins (RBPs, such as hnRNPA2B1 or SYNCRIP) that recognize distinct sorting sequences (e.g., hEXO‐motifs), ensuring their targeted incorporation into the invaginating endosomal membrane during multivesicular body (MVB) maturation [36, 37]. Protected within the genuine exosomal lipid bilayer, these macromolecular noncoding elements, such as lnc‐H19, lnc‐ARSR, and the exonuclease‐resistant covalently closed loop architectures of circ‐FOXO3, are effectively shielded from systemic ribonuclease degradation over long physiological distances [38, 39]. Upon internalization by distant target organs, these genuinely encapsulated long exRNAs act as powerful competitive endogenous RNAs (ceRNAs) or molecular sponges, driving profound transcriptional and epigenetic remodeling of the host microenvironment [40].

This definitive metrological segregation highlights that the tumor secretome operates not through a uniform vesicular entity, but via a highly coordinated, heterogeneous division of labor where nonvesicular carriers dominate acute small RNA kinetics, while true membrane‐bound exosomes specialize in the long‐range transit of highly stable macromolecular RNA complexes.

#### 2.2.2 The Proteomic Landscape

Concurrently with the transcriptomic reassessment, the proteomic configuration of cancer‐derived extracellular vesicles has been rigorously refined. Modern quantitative proteomics dictates that the vesicular proteome must be characterized not as a disordered bulk aggregate, but through its strict topological architecture, segregating proteins into a highly conserved structural endosomal core, vesicular surface receptors, and intraluminal soluble factors. Crucially, in strict alignment with high‐resolution immuno‐isolation benchmarks [41], traditional cytosolic contaminants such as canonical metabolic glycolytic enzymes (e.g., GAPDH) and general cytoskeletal elements have been formally declassified as genuine exosomal constituents. Instead, the verified proteome comprises molecules heavily enriched through active, endosomal sorting systems (e.g., the ESCRT machinery). The structural core comprises universal tetraspanins (CD63, CD9, CD81), endosomal trafficking proteins (ALIX, TSG101), heat shock proteins (HSP70, HSP90), and specialized membrane microdomain scaffolders (Annexins and Flotillins) [42]. Beyond these baseline characterization markers, the variable oncogenic proteome represents the true functional weaponry of the tumor, operating via two primary topological axes:

##### 2.2.2.1 Axis A: Surface‐Displayed Receptors and Immunosuppressive Ligands

Malignant vesicles actively display full‐length, biologically active receptor tyrosine kinases (RTKs) and checkpoint ligands on their outer lipid bilayers, allowing them to engage in direct membrane‐to‐membrane ligation with recipient host cells. A classic pan‐cancer paradigm is the selective sorting and shedding of constitutively active oncogenic receptors, such as the EGFRvIII mutant in high‐grade neuroectodermal malignancies or full‐length EGFR in systemic carcinomas [43]. Upon horizontal transfer via membrane fusion to receptor‐negative bystander cells, these exogenous RTKs instantly integrate into the host plasma membrane, activating downstream mitogen‐activated protein kinase (MAPK) and PI3K/Akt pathways to initiate a collective, microenvironmental proliferative surge. Simultaneously, tumor vesicles leverage this surface‐display architecture to orchestrate systemic immune evasion. Cancer exosomes could expose high‐density immune checkpoint ligands, notably programmed death‐ligand 1 (PD‐L1) and Fas ligand (FasL), on their extracellular surfaces. Circulating exosomal PD‐L1 acts as a powerful decoy that intercepts and binds directly to PD‐1 receptors on surveillance T cells within the vascular channels [44, 45, 46]. This high‐affinity ligation induces robust systemic T‐cell exhaustion and apoptosis long before physical local invasion occurs, neutralizing host adaptive immunity on an organism‐wide scale.

##### 2.2.2.2 Axis B: Encapsulated Luminal Signaling Factors

Shedding contaminating structural debris, the inner luminal core of genuine exosomes specifically encapsulates highly specialized, nonstructural soluble cytokines and growth factors that are delivered directly into the cytoplasm of destination cells [47]. A prominent mechanism is directed by exosomal macrophage migration inhibitory factor (MIF) [48, 49]. When sequestered within the exosomal lumen, MIF is completely shielded from circulating neutralizing antibodies or extracellular proteolytic degradation. Upon targeted internalization by remote resident cells, such as Kupffer cells in hepatic niches or specialized stromal cells in systemic matrices, vesicular MIF activates a localized fibrotic and inflammatory cascade. This molecular delivery heavily upregulates transforming growth factor‐beta secretion and fibronectin deposition, establishing the hyperinflammatory, myeloid‐rich sanctuary required for circulating tumor cells to successfully colonize distant target organs. By stratifying the proteome into surface‐active ligands that trigger immediate transmembrane cascades and luminal factors that drive intracellular niche remodeling, this topological framework explains how tumor vesicles execute precise, multitiered pathogenic programs across both localized and systemic distances.

#### 2.2.3 Lipids, Metabolites, and DNA Fragments

While nucleic acids and proteins dominate functional oncological literature, the lipidomic and metabolomic matrix of the tumor secretome plays an equally critical role in structural stability, cellular uptake, and microenvironmental rewiring [50]. However, in strict accordance with the landmark molecular reassessment of vesicle components, a rigorous distinction must be maintained between the genuine lipid bilayer of membrane‐bound exosomes and the co‐purified nonvesicular macromolecular carriers that contain stable metabolic enzymes and genomic DNA fragments.

##### 2.2.3.1 The Exosomal Lipid Bilayer

The genuine exosomal membrane is markedly enriched in rigid, tightly packed lipid species compared with the donor cell plasma membrane, featuring exceptional concentrations of sphingomyelin, cholesterol, phosphatidylserine (PS), and saturated fatty acids [51, 52]. This specialized lipidomic signature provides extreme physical rigidity and chemical stability, acting as a structural shield that allows exosomes to survive harsh, ribonuclease‐rich extracellular matrices and navigate hydrodynamic shear stress within the systemic circulation over long distances [53]. Beyond structural shielding, this lipid bilayer actively dictates receptor‐cell signaling through precise membrane topology. Under homeostatic conditions, PS is strictly sequestered within the inner leaflet of the plasma membrane. However, during endosomal maturation and MVB shedding in malignant cells, PS undergoes robust externalization to the outer leaflet of the exosomal membrane. This outer‐surface exposure of PS operates across diverse organ systems as a universal, high‐affinity “eat me” signal, facilitating rapid clathrin‐mediated endocytosis, macropinocytosis, or direct membrane fusion with recipient stromal and endothelial populations, thereby accelerating the internalization of internal macromolecular cargos [54, 55].

##### 2.2.3.2 The Metabolomic and DNA Misassignment

Conversely, high‐resolution immuno‐isolation and enzymatic profiling have fundamentally declassified core metabolic enzymes and genomic DNA as intrinsic components of the internal exosomal lumen. Rigorous metrological audits confirmed that critical glycolytic enzymes, including pyruvate kinase M (PKM), lactate dehydrogenase A (LDHA), and enolase 1 (ENO1), are completely excluded from pure, immuno‐isolated endocytic exosomes [56]. Instead, these active metabolic enzymes are securely encapsulated within the nonmembranous, protein‐dense exomeres, where they act as mobile fuel packets. When released into the nutrient‐deprived TME, these exomeric metabolic enzyme complexes horizontally propagate the Warburg effect by directly synthesizing ATP and lactate in the extracellular space, sustaining stromal subversion independently of vesicle fusion [57]. Similarly, the historical assignment of double‐stranded genomic DNA (dsDNA) to the internal exosomal lumen has been comprehensively disproven. High‐resolution analysis demonstrates that the release of large genomic dsDNA fragments is an exosome‐independent process driven by altered autophagy and MVB microfission; these DNA fragments are physically complexed externally with circulating Supermeres or extracellular histones rather than enclosed within lipid bilayers [58]. Nevertheless, because this extracellular dsDNA pool spans the entire nuclear genome and accurately reflects the primary tumor's original mutational landscape, safeguarding critical driver mutations such as BRAF V600E, KRAS, and TP53, it remains an invaluable, highly stable template for blood‐ and cerebrospinal fluid‐based liquid biopsy platforms [59, 60, 61]. By strategically reassigning metabolic machinery to exomeres and genomic DNA to supermeric/nonvesicular fractions, this pan‐cancer synthesis provides a scientifically accurate blueprint of the tumor secretome, ensuring that therapeutic and diagnostic targeting platforms are engineered against verified molecular carriers.

### 2.3 Structural and Size Heterogeneity

As high‐resolution asymmetric‐flow field‐flow fractionation (AF4) and super‐resolution imaging technologies have matured, the conceptual definition of tumor‐derived extracellular vesicles has expanded from a homogeneous population of lipid‐bilayer spheres into a highly complex spectrum of nanoparticle heterogeneities. Historically, the generic term “exosome” was universally applied to all small extracellular vesicles (sEVs) spanning 40 to 150 nm in diameter. However, state‐of‐the‐art multi‐omic analyses have revealed that malignant cells simultaneously secrete distinct subpopulations of both membranous and nonmembranous nanoparticles, each governed by independent intracellular sorting machineries and possessing distinct biological repertoires within the pan‐cancer landscape [62].

Physically, canonical sEVs are now rigorously divided into Large Exosomes (Exo‐L, 90–150 nm) and Small Exosomes (Exo‐S, 40–90 nm). While both subpopulations retain the classic lipid‐bilayer structure decorated with tetraspanins (CD9, CD63, and CD81), their functional payloads are strictly segregated. Exo‐L populations are heavily enriched in signal transduction proteins associated with cell mitotic machinery and late endosomal pathways, whereas Exo‐S fractions selectively sequester specific small noncoding RNAs, metabolic enzymes, and processing machinery like Argonaute proteins, suggesting that Exo‐S acts as the primary vehicle for horizontal gene silencing and rapid phenotypic reprogramming in recipient stromal cells [63].

The most revolutionary paradigm shift in cancer biology, however, is the discovery of distinct classes of nonmembranous ENPs secreted abundantly by systemic tumors: Exomeres (∼35 nm) and Supermeres (∼25 nm) [64, 65]. Unlike canonical exosomes, Exomeres and Supermeres are completely devoid of a protective lipid bilayer membrane, yet they possess an exceptional structural stability that allows them to survive the proteolytic and nucleolytic conditions of the systemic circulation. Mechanistically, these ENPs are not passive cellular debris; they are actively generated through specialized, nonclassical secretory pathways and serve as the absolute concentrated currency for tumor metabolic rewiring and systemic immune evasion. For instance, across multiple solid tumors, Exomeres are found to be highly enriched in structural metabolic enzymes driving glycolysis, the hexosamine pathway, and alcohol metabolism, functioning as circulating metabolic catalysts that precondition peripheral stroma. Meanwhile, Supermeres exhibit an even higher molecular density than exomeres, encapsulating critical oncogenic drivers such as extracellular MET receptor tyrosine kinases, EGFR signaling components, and high concentrations of targetable miRNAs like miR‐1246 [66]. Furthermore, Supermeres are major carriers of extracellular RNA‐binding proteins and essential glycolytic regulators, allowing them to travel deeper into compliant tissue matrices than larger membranous exosomes [67]. Recognizing this intricate structural heterogeneity within the tumor secretome is fundamentally critical; it explains how a single malignant clone can simultaneously dispatch distinct nano‐sized agents to execute localized synaptic remodeling, metabolic hijacking, and systemic immunological paralysis in parallel.

## 3 Exosomal Mechanisms in the Tumor Microenvironment

The malignant progression of systemic tumors relies heavily on their ability to dynamically alter the host stroma, transforming a restrictive physiological environment into a permissive, tumor‐supportive microenvironment. Across a broad pan‐cancer landscape, tumor‐derived exosomes function as the primary currency for this microenvironmental bribery [68]. By delivering specialized molecular cargo to local and distant nonmalignant cells, these vesicles initiate a cascade of stromal remodeling events that orchestrate angiogenesis, metabolic reprogramming, and the preparation of distant metastatic soils. Understanding these universal exosomal mechanisms across systemic malignancies provides the essential biochemical baseline required to appreciate how conserved extracellular pathways are systematically hijacked by distinct tumor types, including those within specialized organ niches.

### 3.1 Induction of Angiogenesis and Vascular Remodeling

Conquering the diffusion limits of oxygen and nutrients is a fundamental hurdle for any growing solid tumor. To initiate neo‐angiogenesis, malignant cells secrete exosomes that directly reprogram local endothelial cells into a pro‐angiogenic state (Figure 2). In non–small cell lung cancer (NSCLC) and colorectal cancer (CRC), hypoxic tumor cells significantly accelerate their exosome production, packaging high levels of vascular endothelial growth factor (VEGF) and basic fibroblast growth factor (bFGF) on the vesicular outer membrane, allowing for clustered, high‐affinity ligation of VEGFR and FGFR on recipient endothelial cells [69, 70, 71]. Concurrently, exosomal noncoding RNAs play an intracellular role after uptake. For instance, breast cancer‐derived exosomal miR‐9 and miR‐155 target endogenous inhibitors of angiogenesis within endothelial cells, such as SOCS1 and FOXO3a, triggering the constitutive activation of the JAK/STAT and pro‐angiogenic signaling cascades [72, 73]. This horizontal transfer induces endothelial cell proliferation, migration, and tube formation. Furthermore, systemic tumor exosomes transport matrix metalloproteinases (e.g., MMP‐2, MMP‐9, and a disintegrin and metalloproteinase with thrombospondin motifs 1 [ADAMTS1]), which actively degrade the extracellular matrix (ECM) and basement membrane surrounding existing vessels, clearing the physical path for sprouting capillaries and facilitating tumor cell intravasation into the bloodstream [74]. Furthermore, the angiogenic switch mediated by exosomal communication is not restricted to solid epithelial malignancies but represents a deeply conserved survival strategy in hematological and highly metastatic neuroectodermal tumors. In multiple myeloma (MM), a classic hematological malignancy of the bone marrow niche, tumor‐derived exosomes actively encapsulate high levels of pro‐angiogenic cytokines, including interleukin‐6 (IL‐6), VEGF, and the oncogenic miRNA miR‐135b [75, 76]. Upon internalization by bone marrow endothelial cells, exosomal miR‐135b directly targets factor‐inhibiting HIF‐1 (FIH‐1), thereby accelerating the transcriptional activity of hypoxia‐inducible factor‐1 (HIF‐1) under normoxic conditions and triggering an aggressive endothelial sprouting cascade within the bone marrow sanctuary [77]. Concurrently, in cutaneous melanoma, highly metastatic primary lesions utilize waves of circulating exosomes enriched with the transmembrane receptor MET (hepatocyte growth factor receptor) to reprogram local and regional lymphatic structures. These melanoma‐derived exosomes selectively home to sentinel lymph nodes, where they degrade endothelial tight junctions via vesicular MMPs and induce specialized lymphatic endothelial cell proliferation. This targeted lymphatic vascular remodeling successfully establishes a regional premetastatic vascular niche, clearing the physical and metabolic routes for early melanoma systemic dissemination [78].

**FIGURE 2:**
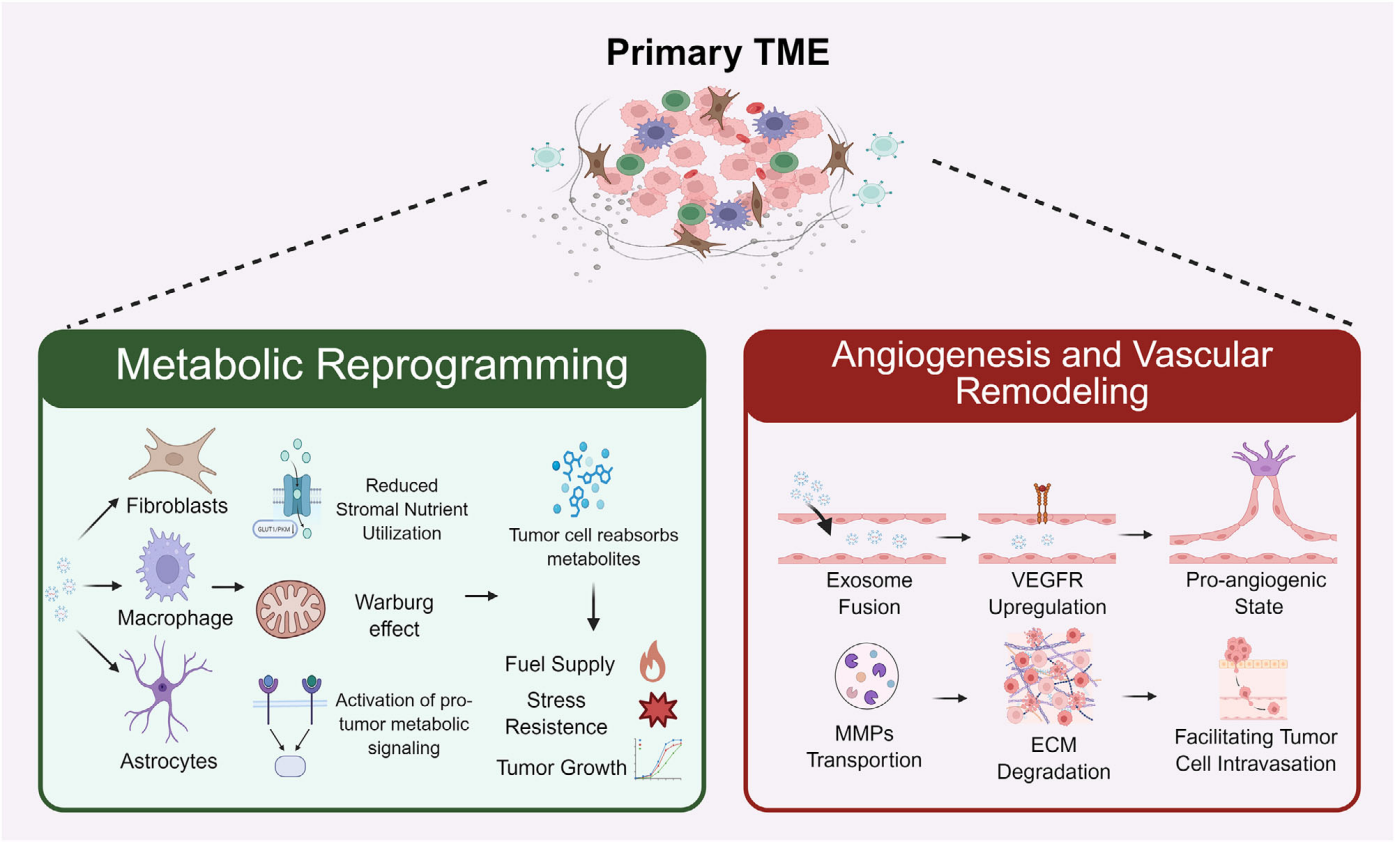
Tumor exosome‐mediated remodeling of the primary tumor microenvironment. Tumor‐derived exosomes remodel the primary tumor microenvironment (TME) by reprogramming stromal metabolism and promoting angiogenesis. Exosomal cargos alters fibroblasts, macrophages, and astrocytes to enhance Warburg‐like metabolism, reduce stromal nutrient consumption, and increase metabolite recycling to support tumor growth and stress resistance. In parallel, exosomes activate endothelial pro‐angiogenic signaling, upregulate VEGFR‐related pathways, transport matrix‐degrading enzymes, and facilitate extracellular matrix remodeling, thereby promoting vascular remodeling and tumor cell intravasation. *Source*: Created with BioRender.com.

### 3.2 Metabolic Reprogramming of the Stromal Landscape

Beyond structural vascular remodeling, tumor‐derived exosomes act as powerful metabolic instructors, orchestrating a widespread energetic rewiring known as the Warburg effect across the stromal landscape (Figure 2) [79]. Malignant cells, characterized by high glycolytic rates, package key glycolytic enzymes and metabolic modulators into exosomes to force neighboring nonmalignant cells into a state of metabolic subservience [80]. A major paradigm is observed in breast cancer, where tumor exosomes transport high concentrations of miR‐122 to surrounding niche cells, such as lung fibroblasts and brain astrocytes [81]. Once internalized, miR‐122 suppresses PKM and glucose transporter 1 (GLUT1) expression in these stromal cells, drastically decreasing their nutrient utilization. This systemic suppression allocates an abundance of extracellular glucose exclusively for the neighboring consumption of the primary tumor, fueling its unhindered proliferative demands under nutrient‐deprived conditions. Conversely, in cancers like gastric adenocarcinoma and pancreatic ductal adenocarcinoma (PDAC), exosomes actively transfer active metabolic enzymes, including hexokinase 2 (HK2) and PKM2, directly into resident fibroblasts [82, 83]. This enzymatic transfer converts resting fibroblasts into cancer‐associated fibroblasts (CAFs), forcing them to undergo aerobic glycolysis and intensely secrete high‐energy metabolites, such as pyruvate and lactate. These metabolites are then re‐absorbed by the primary tumor cells via monocarboxylate transporters (MCTs) to fuel the mitochondrial tricarboxylic acid (TCA) cycle, establishing a reciprocal, exosome‐driven metabolic symbiosis that protects the tumor from oxidative stress and nutrient deprivation. Similarly, exosome‐driven metabolic rewiring aggressively operates within the specialized stromal environments of the urogenital tract. In clear cell renal cell carcinoma (ccRCC), hypoxic tumor cells highly enrich their exosomal cargo with hypoxia‐induced lncRNAs, specifically lnc‐ARSR [84]. When these vesicles are internalized by adjacent tumor‐associated macrophages (TAMs) or resting renal fibroblasts, lnc‐ARSR acts as a molecular sponge to sequester endogenous miR‐34a and miR‐449a, leading to the robust upregulation of AXL and c‐MET signaling axes within the stroma. This horizontal epigenetic transmission forces target stromal cells to prioritize the pentose phosphate pathway and aerobic glycolysis over oxidative phosphorylation, actively secreting a high‐energy metabolic surplus of glutamine and pyruvate into the extracellular matrix. This local metabolic wealth is rapidly recaptured by nutrient‐starved renal carcinoma cells to fuel their macromolecular synthesis, illustrating a universal, cross‐cancer paradigm where exosomal delivery forces diverse stroma to serve as autonomous metabolic factories for the primary tumor.

### 3.3 Premetastatic Niche (PMN) Formation and Organotropic Homing

The most complex and long‐range manifestation of pan‐cancer exosomal communication is the establishment of the premetastatic niche (PMN) (Figure 3). Long before malignant cells physically escape the primary site, they shed waves of systemic exosomes into the bloodstream to prepare distant, future organs for metastatic colonization. This distant soil preparation is highly organized and organotropic, determined by specific molecular ZIP codes on the exosome surface [85]. Landmark studies have demonstrated that exosomal integrins (ITGs) dictate the organotropic homing of systemic tumors: breast cancer exosomes enriched with integrins α_6_β_4_ and α_6_β_1_ specifically adhere to laminin‐rich lung resident fibroblasts, promoting pulmonary metastasis, whereas pancreatic and gastric cancer exosomes displaying integrin α_v_β_1_ selectively bind to fibronectin on Kupffer cells, driving hepatic metastasis [86, 87, 88]. Upon homing to these target tissues, exosomal cargo activates resident stromal populations; for example, PDAC exosomal macrophage migration inhibitory factor (MIF) induces transformational signaling in Kupffer cells, triggering the secretion of transforming TGF‐β. This fibrotic cascade upregulates fibronectin deposition and recruits bone marrow‐derived CD11b^+^ myeloid cells to the site, creating a hyper‐inflammatory, immunosuppressive localized environment. This newly formed PMN acts as a protective sanctuary, ensuring that when circulating tumor cells (CTCs) eventually arrive, they can immediately adhere, survive immune surveillance, and successfully initiate macrometastatic growth.

**FIGURE 3:**
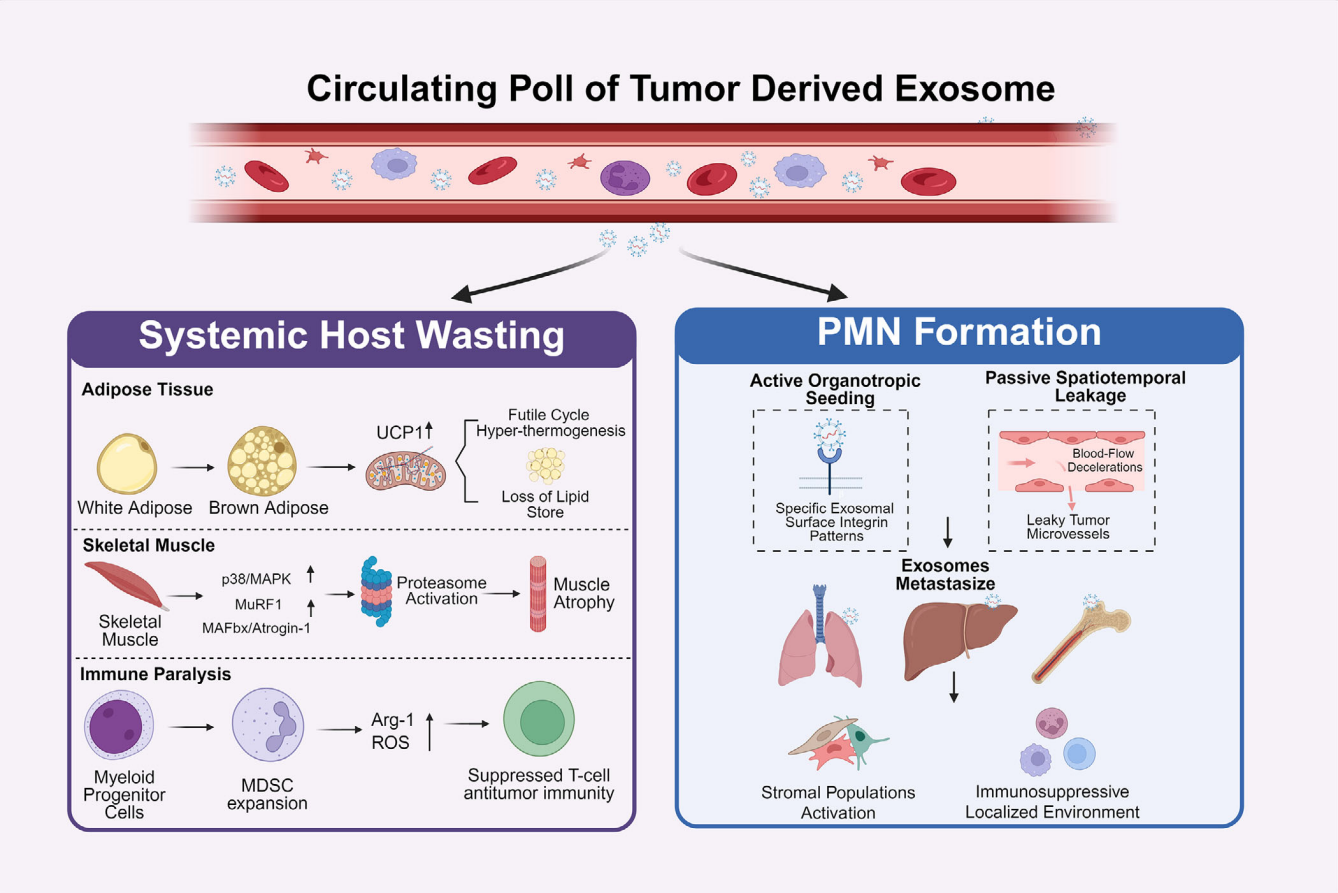
Systemic effects of circulating tumor‐derived exosomes in host wasting and premetastatic niche formation. Circulating tumor‐derived exosomes mediate long‐range remodeling of host tissues by driving systemic wasting and preparing distant metastatic sites. Exosomal cargos promote adipose browning, skeletal muscle proteolysis, and myeloid‐derived suppressor cell expansion, resulting in lipid depletion, muscle atrophy, and suppressed T‐cell antitumor immunity. In parallel, tumor exosomes contribute to premetastatic niche (PMN) formation through both active organotropic homing, mediated by specific exosomal surface integrins, and passive spatiotemporal leakage caused by vascular permeability and blood‐flow deceleration, ultimately supporting stromal activation, immunosuppression, and metastatic colonization. *Source*: Created with BioRender.com.

In conclusion, the functional repertoire of tumor‐derived exosomes within the systemic microenvironment underscores their role as indispensable architects of malignancy. By simultaneously orchestrating localized endothelial sprouting, forcing stromal cells into metabolic submission, and systematically preparing distant target organs for colonization via integrin‐mediated tropism, exosomes provide a unified mechanism for pan‐cancer progression. These conserved pathways highlight a fundamental biological truth: tumor survival depends entirely on the continuous subversion of normal host tissue. Recognizing these universal principles establishes the critical conceptual foundation necessary to explore how these very same vesicular strategies are uniquely modified, refined, and deployed when tumors encounter the formidable, highly specialized anatomical and immunological barriers of the central nervous system.

### 3.4 Systemic Host Wasting: Exosome‐Driven Cachexia and Immune Paralysis

Beyond shaping the local tumor microenvironment and sculpting distant premetastatic niches, tumor‐derived exosomes operate as systemic drivers of host metabolic and immunological collapse, orchestrating the severe clinical syndrome known as cancer cachexia (Figure 3) [89]. Cancer cachexia, characterized by the progressive and involuntary wasting of skeletal muscle and adipose tissue, was historically attributed to passive nutrient consumption by the tumor or generalized systemic inflammation. However, current pan‐cancer paradigms demonstrate that circulating exosomes act as targeted endocrine‐like messengers that actively reprogram central and peripheral host tissues to force systemic energy dissipation and tissue catabolism [90, 91, 92].

In adipose tissue, solid tumors such as pancreatic, colorectal, and gastric adenocarcinomas deploy circulating exosomes to hyper‐accelerate adipose tissue browning. Under physiological conditions, energy‐storing white adipose tissue (WAT) maintains lipid homeostasis, while brown adipose tissue (BAT) drives thermogenesis [93, 94]. Malignant vesicles aggressively disrupt this balance by delivering specialized chronotropic and metabolic payloads, notably parathyroid hormone‐related protein (PTHrP) and specific miRNAs such as miR‐21 and miR‐146a [95, 96, 97]. Upon uptake by peripheral adipocytes, exosomal miR‐21 directly downregulates the TLR7/NF‐κB downstream negative regulators or directly targets PTEN, triggering the robust transcriptional activation of uncoupling protein 1 (UCP1) within the mitochondrial matrix. This ectopic UCP1 upregulation uncouples mitochondrial oxidative phosphorylation from ATP synthesis, forcing white adipocytes to rapidly shift toward a high‐energy‐burning brown phenotype [94, 98]. This exosome‐driven browning cascade triggers extensive lipolysis and futile cycle hyperthermogenesis, rapidly depleting the host's lipid reserves. Simultaneously, circulating tumor exosomes cross into the skeletal muscle architecture to drive myofibrillar protein degradation and muscle wasting. Vesicular payloads rich in Hsp70/90 or miR‐21 engage TLR7 on the surface of murine and human myoblast membranes, inducing a chronic, localized apoptotic cascade [99, 100]. In parallel, this signaling activates the classic p38/MAPK pathway, which heavily upregulates the expression of muscle‐specific E3 ubiquitin ligases, including muscle RING finger 1 (MuRF1) and muscle atrophy F‐box (MAFbx/Atrogin‐1) [101]. The resultant ubiquitin‐proteasome hyperactivation selectively degrades core myofibrillar structural components, such as myosin heavy chain proteins, leading to profound cachectic muscle atrophy and physical frailty.

Finally, this systemic wasting is complemented by exosome‐driven immune paralysis at the bone marrow level. Circulating tumor exosomes travel to the hematological sanctuary, where they reprogram myeloid progenitor cells to bypass conventional dendritic cell or macrophage differentiation. Instead, they force the massive expansion and systemic mobilization of myeloid‐derived suppressor cells (MDSCs). These newly generated MDSCs flood the systemic circulation and home back into the tumor matrix, where they release excessive quantities of arginase‐1 (Arg‐1) and reactive oxygen species (ROS), profoundly paralyzing host T‐cell‐mediated antitumor immunity [102, 103]. By linking local oncogenesis to systemic adipose browning, myofibrillar degradation, and hematological subversion, exosomal communication establishes a devastating organism‐wide hijacking of host survival infrastructure.

### 3.5 Conceptual Debate: Active Organotropic Seeding versus Passive Spatiotemporal Leakage

While the preceding paradigms conceptualize tumor‐derived exosomes as high‐precision, “guided missiles” capable of autonomously seeking and remodeling distant premetastatic niches, a critical and rigorous evaluation of in vivo vesicle dynamics reveals a profound biological controversy. The pervasive narrative of absolute organotropism, largely driven by the discovery of specific exosomal surface integrin patterns (e.g., α_6_β_4_ driving lung tropism and α_v_β_5_ driving liver tropism) protecting directional docking, must be carefully weighed against the stark realities of systemic clearance kinetics [10, 104]. Quantitative biodistribution tracking indicates that upon entering the systemic circulation, more than 90% of injected or endogenously shed exosomes are rapidly captured and eliminated within minutes by the reticuloendothelial system (RES), primarily through phagocytosis by resident Kupffer cells in the liver and red pulp macrophages in the spleen [105, 106]. Consequently, the literal concentration of intact tumor vesicles reaching distal target organs like the brain or bone marrow is exceptionally diluted, challenging the anthropomorphic assumption of highly localized, active “seeding” at the single‐nanoparticle level.

This physiological bottleneck strongly suggests that exosome accumulation in distant niches may be governed equally by passive spatiotemporal leakage. According to this counter‐paradigm, primary tumors induce microvascular hyperpermeability, localized endothelial cell fenestrations, and blood‐flow decelerations within specific capillary beds via the systemic release of circulating cytokines [107, 108, 109, 110]. This systemic vascular compromise creates structural traps, causing passing exosomes to undergo passive fluidic stagnation and mechanical entrapment within loose extracellular matrices. Therefore, what appears to be molecularly targeted docking may simply represent a stochastic accumulation where vesicles leak into predamaged microenvironments. Dissecting whether PMN evolution is initiated by precision molecular cargo delivery or accelerated by passive hemodynamic entrapment remains a defining frontier in extracellular vesicle biology [111]. Future translation must transcend this dichotomy by developing rigid, real‐time intravital quantitative tracking to map the precise ratios of active ligated endocytosis versus passive paracellular extravasation across diverse host organs.

## 4 Tissue‐Specific Heterogeneity and Spatial Complexity of the Tumor Microenvironment

The systemic macroevolution of pan‐cancer progression dictates that while the intracellular cascades of malignant transformation share highly conserved genetic drivers across diverse lineages, the ultimate functional deployment of these oncogenic programs is strictly governed by the physiological, architectural, and anatomical realities of the host organ (Figure 4). This geographic determinism establishes a multidimensional matrix of tissue‐specific heterogeneities, shattering the archaic view of a uniform TME [112]. Across the human body, solid and hematological malignancies must continuously adapt their exosomal communication strategies to breach unique local biological barriers and subvert organ‐specific stromal cells. For instance, primary and metastatic tumors in the bone marrow must navigate a highly hypoxic, mineralized niche populated by specialized osteoclasts and endosteal cells, while lesions in the hepatic parenchyma encounter the distinct filtering mechanics of fenestrated sinusoidal endothelial cells and resident Kupffer populations [113, 114, 115]. Consequently, tumor‐derived exosomes cannot function through a generalized, one‐size‐fits‐all signaling paradigm; instead, their surface tropism and encapsulated cargo molecular blueprint are dynamically tailored to decipher, compromise, and reprogram the unique biophysical and immunotolerant constraints of each target tissue [116].

**FIGURE 4:**
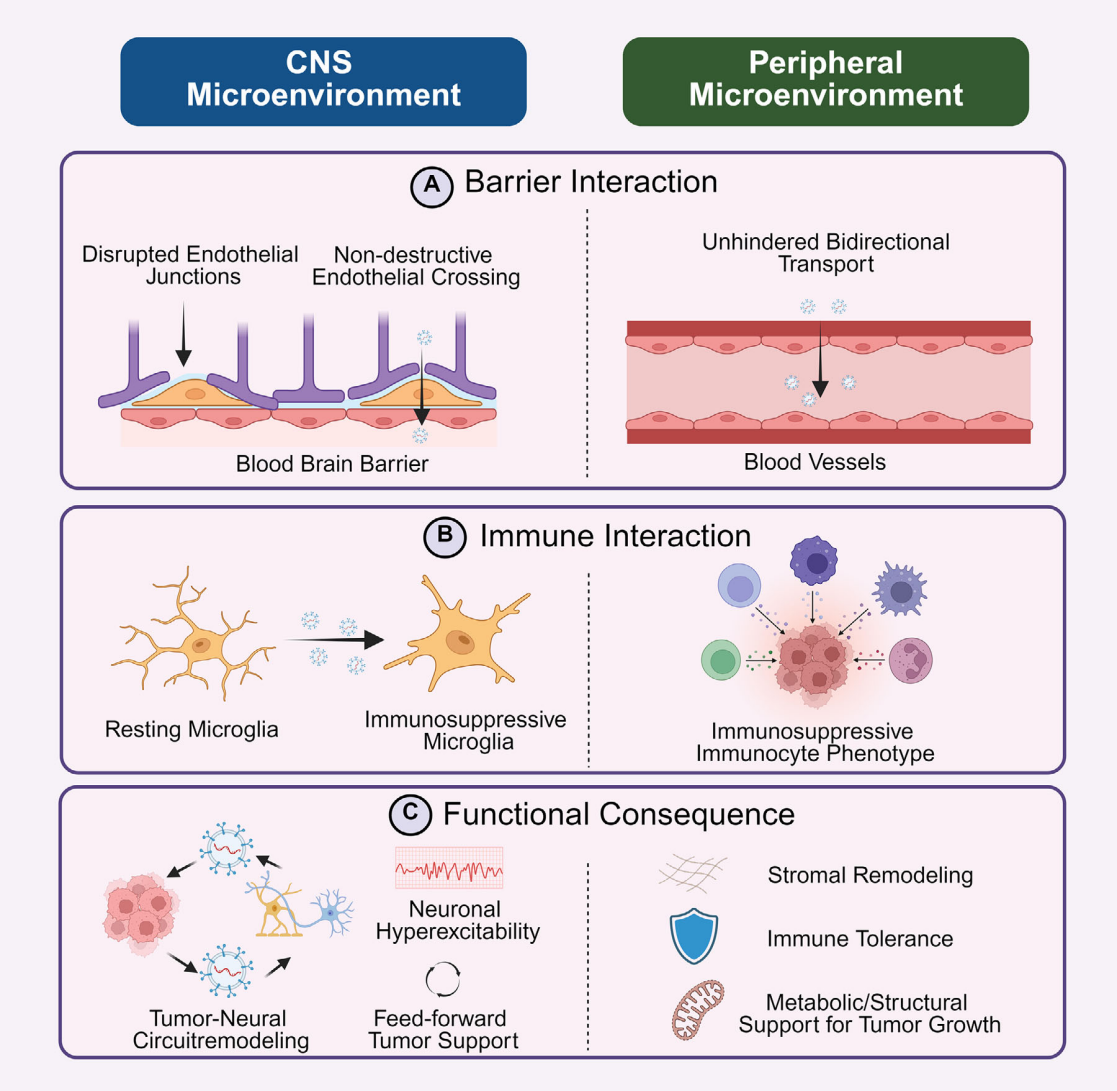
Tissue‐specific adaptation of tumor exosome signaling in CNS and peripheral microenvironments. Tumor‐derived exosomes exert distinct biological effects depending on the anatomical and stromal context. In the CNS, exosomes must interact with the blood–brain barrier through tight‐junction disruption or nondestructive endothelial transcytosis, reprogram resident microglia into immunosuppressive phenotypes, and promote tumor–neural circuit remodeling that drives neuronal hyperexcitability and feed‐forward tumor support. In contrast, peripheral tumor exosomes undergo relatively unhindered vascular transport, induce broader immunosuppressive leukocyte phenotypes, and support tumor progression through stromal remodeling, immune tolerance, and metabolic or structural support. *Source*: Created with BioRender.com.

Among these diverse geographic paradigms, the CNS represents one of the most sophisticated and highly isolated organizational examples of tissue‐specific complexity, serving as a premier biological model to study the limits of exosome‐mediated horizontal communication. The brain parenchyma presents an array of extreme physical and immunological barriers that do not exist in peripheral epithelial or mesenchymal matrices, notably the absolute lack of traditional fibronectin or loose collagen networks, which are replaced instead by a dense matrix of glycosaminoglycans and hyaluronic acid [117, 118]. To successfully colonize or originate within this unique sanctuary, malignant vesicles must upgrade their molecular artilleries from simple matrix degradation to highly advanced cellular engineering. Dissecting the CNS microenvironment as a defining example of tissue‐specific complexity offers profound translational insights for the entire pan‐cancer landscape. It illustrates how universal extracellular vesicle signaling networks are uniquely modified, refined, and deployed when confronting the most formidable physiological constraints of the human body, specifically manifested through the targeted crossing of the blood–brain barrier (BBB), the phenotypic subversion of resident microglia, and the highly specialized integration of malignant clones into active synaptic neural circuits [119, 120].

### 4.1 Biological Crossing and Subversion of the Blood–Brain Barrier

In peripheral systemic malignancies, tumor‐derived exosomes travel through blood vessels that possess varying degrees of physiological fenestration and permeability, allowing for unhindered bidirectional transport between the interstitial fluid and the circulatory system [121]. In sharp contrast, the CNS example is protected by the BBB, a highly restrictive, specialized neurovascular unit comprising nonfenestrated brain capillary endothelial cells (BCECs), continuous endothelial tight junctions (TJs), pericytes, and a dense covering of astrocytic end‐feet. Under physiological conditions, this formidable barrier restricts the paracellular diffusion of almost all hydrophilic macromolecules and circulating cells, protecting brain homeostasis but simultaneously posing a severe logistical challenge for high‐grade gliomas attempting to orchestrate systemic communication [122].

To circumvent this anatomical restriction, glioma‐derived exosomes (GDEs) utilize highly evolved mechanisms to selectively compromise or cross the BBB. Unlike systemic tumors that rely on general matrix degradation, GDEs actively package specific noncoding RNAs, notably miR‐181a, miR‐21, and miR‐132, which target the core structural proteins of endothelial tight junctions [123]. Upon internalization by BCECs, exosomal miR‐181a directly downregulates the expression of Claudin‐5, Occludin, and Zonula Occludens‐1 [124, 125]. This targeted degradation leads to a significant loss of structural integrity at endothelial cell‐cell contacts, inducing paracellular leakiness and allowing GDEs to freely pass through the structurally weakened barrier. Furthermore, GDEs exploit advanced transcellular pathways to cross intact endothelial walls without destroying them. This transcytosis is frequently mediated by the interaction between exosomal surface tetraspanins (such as CD9 and CD63) or customized ligands and specific endocytic receptors (e.g., low‐density lipoprotein receptor‐related protein 1 [LRP1]) expressed on the luminal surface of BCECs, triggering a specialized caveolae‐mediated endocytosis cascade [126]. This active transport allows GDEs to be internalized, ferried across the endothelial cytoplasm, and successfully exocytosed on the abluminal side into the brain parenchyma.

### 4.2 Divergent Immune Landscapes

The immunological dialogue mediated by exosomes varies dramatically when comparing peripheral systemic organs to the brain parenchyma. In systemic adenocarcinomas, tumor exosomes circulate into highly accessible peripheral immune tissues, such as the spleen, bone marrow, and lymph nodes, where they predominantly interact with circulating monocytes and dendritic cells to induce systemic immunosuppression [127]. In the brain example, however, the primary cellular target for tumor‐derived vesicles is a highly specialized population found nowhere else in the periphery: resident microglia, the innate immune sentinels of the CNS, along with border‐associated macrophages [128, 129].

While peripheral tumor exosomes drive M2‐macrophage polarization through generic cytokine pathways, GDEs hijack specialized, central‐nervous‐system‐specific neural communication axes to force microglia into a highly tumor‐supportive, immunosuppressive state. High‐grade gliomas package specific oncogenic cargo, including miR‐1246, miR‐21‐5p, and signal transducer and activator of transcription 3 (STAT3) proteins, directly into the exosomal lumen [130, 131, 132]. When these vesicles are selectively internalized by resting microglia via phagocytosis, the delivered miR‐1246 directly targets the GSK‐3β/β‐catenin signaling axis, suppressing the pro‐inflammatory M1 phenotype (characterized by the loss of iNOS, IL‐12, and TNF‐α expression) and aggressively upregulating the immunosuppressive M2 phenotype (marked by high expression of Arg‐1, IL‐10, and TGF‐β). This exosome‐driven transformation converts microglia from highly vigilant immune defenders into active protectors of the tumor. These reprogrammed microglia actively synthesize matrix‐degrading enzymes that clear physical paths for glioma invasion and release high concentrations of neurotrophic factors that directly support the primary tumor's survival, representing a profound subversion of immune privilege [133, 134].

### 4.3 Tumor–Neural Circuit Interactions

The most distinct biological feature that isolates neuro‐oncology from all systemic peripheral malignancies is the absolute integration of the tumor into the active electrical and synaptic networks of the host organ. In peripheral cancers like lung, breast, or prostate carcinoma, tumor cells grow within connective or epithelial matrices where they interact with fibroblasts, adipocytes, and immune cells; however, they do not participate in active, synchronized electrophysiological circuitry. In high‐grade gliomas, the primary tumor exists in direct, continuous physical contact with functional neurons and active synaptic networks [135, 136].

Recent breakthroughs in cancer neuroscience have revealed that GDEs serve as the biochemical currency driving this highly specialized tumor‐neural circuit interaction. Glioma cells actively release waves of GDEs that encapsulate neurotransmitter receptors, synaptic plasticity modulators, and explicit miRNAs such as miR‐21 and miR‐451a [137, 138]. When these vesicles are taken up by neighboring healthy neurons, they induce widespread synaptic remodeling and hyperexcitability. Mechanistically, GDEs deliver specific noncoding RNAs that selectively downregulate the expression of glutamate transporters (such as GLT‐1/EAAT2) in surrounding astrocytes and neurons, leading to a toxic accumulation of extracellular glutamate within the synaptic cleft. This persistent glutamate surplus triggers chronic overactivation of neuronal NMDA and AMPA receptors, inducing localized neuronal hyper‐excitability and ultimately driving the clinical presentation of glioma‐associated epilepsy [139, 140]. Critically, this neuronal hyperactivity is not merely a clinical symptom; it establishes a devastating feed‐forward loop. Hyperexcited neurons intensely release specific neurotrophins, notably neuroligin‐3 (NLGN3) and brain‐derived neurotrophic factor (BDNF), into the synaptic space. These neurotrophins act as powerful mitogens that bind back onto glioma cells, accelerating tumor cell proliferation, driving synaptic network integration, and fueling tumor invasion along white matter tracts [141]. This electrophysiological and biochemical symbiosis between malignant cells and active neural pathways represents a landmark mechanism entirely unique to neuro‐oncology, highlighting why generalized pan‐cancer models fail to capture the true clinical nature of brain tumors.

In conclusion, comparing the exosomal communication networks across diverse systemic malignancies highlights that tumor survival depends entirely on subverting local tissue‐specific heterogeneity. The biological reality of the CNS, operating as a primary case study of maximum spatial complexity, demonstrates how conserved vesicle pathways are uniquely elevated to breach the BBB, paralyze resident microglia, and systematically reshape functional neuronal circuitry. Recognizing these tissue‐specific mechanics emphasizes that future therapeutic engineering must abandon generalized systemic drug‐delivery platforms. Instead, precision medicine must leverage these very same specialized organotropic pathways to construct next‐generation, biomimetic therapeutic vehicles calibrated for the distinct physiological parameters of target tissues.

## 5 Clinical Translations: Diagnostic Benchmarks and Therapeutic Engineering

The clinical translation of exosome biology marks a transformative frontier in molecular oncology, offering unprecedented opportunities to shift the paradigms of both noninvasive diagnostics and targeted nanomedicine. Circulating extracellular vesicles function as sensitive barometers for early detection, dynamic molecular subtyping, and longitudinal therapeutic monitoring [142]. However, translating these technological milestones into clinical reality requires a sophisticated navigation of distinct biological fluid compartments, varying anatomical boundaries, and rigorous physical barriers. Rather than operating within a uniform physiological channel, exosome‐based translation must actively adapt to the spatial and microenvironmental heterogeneities of the host organism, integrating fluid‐specific isolation protocols (such as discriminating blood‐borne targets from specialized cerebrospinal fluid matrices) with advanced biomimetic surface engineering to achieve high‐resolution precision oncology [143].

Concurrently, the therapeutic exploitation of the tumor secretome has evolved into a highly integrated, multidimensional discipline. This clinical translation spans two primary pharmacological axes: an offensive paradigm that leverages engineered, cargo‐loaded natural or synthetic biomimetic vesicles to overcome formidable biological barriers for targeted drug delivery, and a defensive paradigm designed to actively intercept, clear, or pharmacologically inhibit endogenously secreted malignant vesicles that drive systemic immunosuppression and niche remodeling [144]. This section systematically evaluates the current diagnostic benchmarks across diverse biofluids and molecular cancer subtypes, details the engineering strategies governing both biomimetic vehicle delivery and extracorporeal therapeutic interception, and provides a comprehensive analysis of the active global clinical trial registry.

### 5.1 Multifluid Liquid Biopsy and Molecular Subtyping

The clinical utilization of the tumor secretome for liquid biopsy relies on the capacity of extracellular cargo profiling to stratify malignancies into distinct molecular subtypes, directly complementing or replacing conventional tissue biopsies (Figure 5A). Across the pan‐cancer landscape, this diagnostic potential is fundamentally dictated by geographic determinism—the specific anatomical site of the primary tumor determines which biofluid compartment serves as the optimal proximal reservoir for capturing tumor clonal evolution without encountering prohibitive signal‐to‐noise or systemic dilution limitations [145].

**FIGURE 5:**
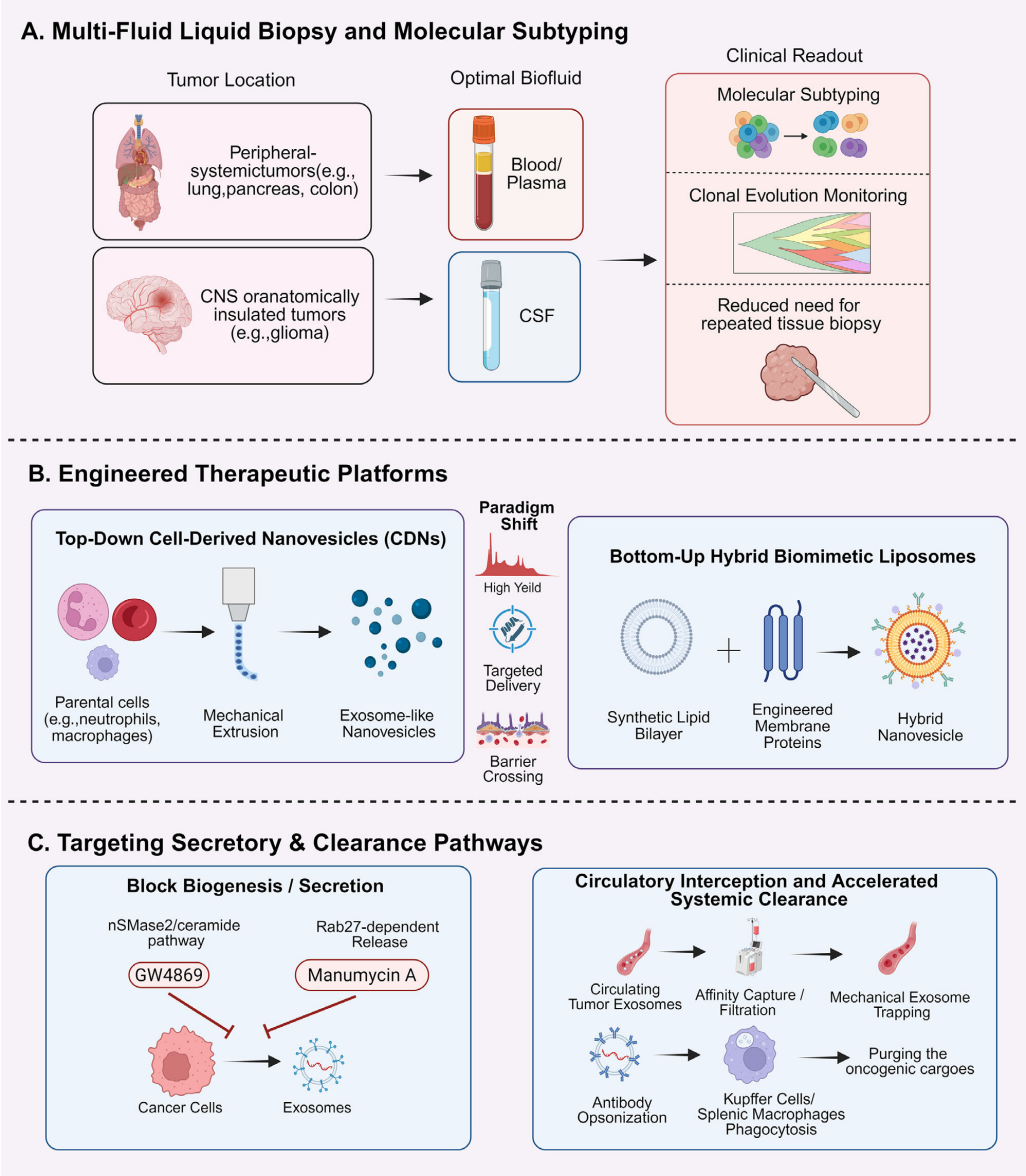
Clinical translation of tumor exosome biology in diagnostics and therapeutic engineering. (A) Multifluid liquid biopsy and molecular subtyping. Tumor anatomical location determines the optimal biofluid for exosome‐based diagnostics. Blood or plasma is suitable for peripheral systemic tumors, whereas cerebrospinal fluid (CSF) provides a more direct reservoir for CNS tumors. Exosomal cargo profiling enables molecular subtyping, clonal evolution monitoring, and may reduce the need for repeated tissue biopsy. (B) Engineered therapeutic platforms. Exosome‐inspired nanomedicines include top‐down cell‐derived nanovesicles generated by mechanical extrusion of parental cells and bottom‐up hybrid biomimetic liposomes constructed from synthetic lipid bilayers and engineered membrane proteins. These platforms improve vesicle yield, targeting precision, barrier crossing, and therapeutic cargo delivery. (C) Targeting secretory and clearance pathways. Malignant exosome communication can be therapeutically disrupted by blocking vesicle biogenesis and secretion, such as through inhibition of the nSMase2/ceramide pathway or Rab27‐dependent release. Circulating tumor exosomes can also be intercepted or cleared by affinity capture, antibody opsonization, macrophage‐mediated phagocytosis, and mechanical exosome trapping. *Source*: Created with BioRender.com.

Rather than relying on a singular vascular channel, modern precision oncology utilizes a multifluid matrix tailored to the anatomical routing of specific tumor beds. For malignancies arising within highly vascularized, compliant systemic environments, the peripheral bloodstream serves as the primary conduit for tumor‐derived EVs. In breast cancer stratification, circulating plasma vesicles function as noninvasive proxies for tissue receptors; cohorts from Luminal A/B subtypes present enrichment of specific mucins and estrogen‐regulated transcripts, whereas HER2‐overexpressing profiles display high‐density, active full‐length HER2 proto‐oncogenes on their bilayers, guiding trastuzumab stratification [146, 147]. Conversely, aggressive Triple‐Negative Breast Cancer (TNBC) sheds a plasma fingerprint dominated by ubiquitinated EGFR, CD44v6, and immunosuppressive miR‐21/miR‐10b clusters [148, 149]. This vascular tracking is mirrored in NSCLC, where plasma vesicles capture driver oncogene mutations (such as EGFR exon 19 deletions or L858R mutations) and selectively encapsulate phosphorylated EGFR proteins and specialized circular RNAs (circ‐CPA4), allowing clinicians to intercept targeted therapy resistance mutations like the secondary T790M gating mutation long before macroscopic radiological progression [150, 151, 152].

However, when tumors are insulated by strict anatomical boundaries or located within specialized tracts, precision subtyping must pivot away from the bloodstream to proximal, compartmentalized biofluids to overcome systemic clearance and blood‐borne dilution. In neuro‐oncology, where the blood–brain barrier severely limits the peripheral vascular yield of GDEs, cerebrospinal fluid (CSF) provides direct, unadulterated fluidic contact with the intracranial parenchyma. CSF‐derived fractions elegantly map the transcriptomic architecture of glioma across its proneural (enriched in PDGFRA alterations and IDH1/2 transcripts), classical (characterized by EGFRvIII transcripts and specialized lipid rafts, alongside co‐purified nonvesicular supermeric fragments carrying chromosome 7 gain/10 loss DNA), and mesenchymal subpopulations (loaded with IL‐6, TIMP1, and surface PD‐L1/FasL) [153, 154].

By sampling this diverse, multifluid matrix, clinicians can capture spatial intratumoral heterogeneity, monitor real‐time clonal evolution, and detect spontaneous phenotype switching (such as the proneural‐to‐mesenchymal transition or chemotherapy‐induced neuroendocrine differentiation) across long physiological distances. This integrated platform ultimately establishes a highly dynamic, comprehensive alternative to repeated, high‐risk tissue biopsies for vulnerable patient cohorts throughout the entire human body.

### 5.2 Preclinical Animal Models and Engineered Therapeutic Platforms

The exceptional biocompatibility, minimal immunogenicity, and natural capacity for cell‐to‐cell fusion render exosomes ideal platforms for next‐generation targeted drug delivery across diverse malignancies. However, wild‐type, unengineered exosomes often suffer from rapid hepatic and splenic clearance mediated by the reticuloendothelial system (RES) and lack tissue‐specific homing capabilities when delivered systemically. To overcome these limitations, preclinical animal modeling across systemic and barrier‐insulated malignancies heavily relies on advanced surface peptide engineering to guide organotropic delivery and evade host surveillance [155] (Table 1).

**TABLE 1:** Preclinical animal studies of exosome‐based therapeutic platforms categorized by pathway and mechanism of action.

Pathway/Targeting mechanism	Engineering strategy or cargo	Tumor model	Mechanism of action	Preliminary findings
αv integrin‑targeted delivery [156, 157, 158]	Exosomes engineered to display iRGD peptide (fused to Lamp2b) on the surface	Orthotopic breast and pancreatic mouse models	iRGD binds tumor endothelial integrins and activates neuropilin‐1‐mediated penetration; enhances intratumoral delivery of therapeutic cargos	Increased tumor accumulation and improved delivery of chemotherapeutic agents including doxorubicin and paclitaxel
nAChR‑mediated transcytosis across the BBB [159, 160, 161, 162]	RVG peptide‑ or T7 peptide‑conjugated exosomes	Orthotopic brain tumor models	RVG interacts with neuronal nicotinic acetylcholine receptors and facilitates receptor‐mediated transcytosis across the BBB	Systemically injected vesicles cross the BBB via receptor‑mediated transcellular transcytosis and deliver siRNA or small‑molecule inhibitors specifically to malignant cells, suppressing tumor growth and extending survival
Exosome biogenesis inhibition [174, 175]	nSMase2 inhibition (GW4869)	Breast, lung, and other solid tumor models	Blocks ceramide‐dependent intraluminal vesicle formation and decreases oncogenic exosome secretion	Reduced tumor‐promoting exosomal signaling and increased response to anticancer therapies
Rab‐dependent secretion inhibition [176, 177]	Rab27a/Rab27b pathway inhibition	Experimental solid tumor models	Prevents MVB docking and exosome release from malignant cells	Reduced circulating tumor exosome levels and impaired metastatic niche formation

#### 5.2.1 Peptide Surface Engineering for Targeted Organotropic Delivery

In systemic tumor models (e.g., orthotopic breast and pancreatic mouse models), exosomes are frequently engineered to display the iRGD peptide (a cyclic tumor‐homing peptide, CRGDKGPDC) fused to the extravesicular loop of the exosomal membrane protein Lamp2b. The iRGD‐engineered exosomes bind with high affinity to α_v_ integrins overexpressed on tumor endothelial cells, triggering a specialized neuropilin‐1‐mediated endocytosis pathway that drastically accelerates deep tumor tissue penetration and enhances the delivery of encapsulated chemotherapeutic agents like Doxorubicin (DOX) or Paclitaxel (PTX) [156, 157, 158].

For applications requiring the crossing of tight endothelial boundaries, the engineering barrier is twice as steep: therapeutic vehicles must possess both the capability to cross intact microvascular junctions and the specificity to selectively home onto target parenchymal cells. To achieve this dual targeting in preclinical models, researchers utilize genetic engineering or chemical conjugation to decorate the exosome surface with the rabies virus glycoprotein (RVG) peptide or the T7 peptide (HAIYPRH). In orthotopic tumor models, systemically injected RVG‐engineered exosomes bind specifically to the nicotinic acetylcholine receptors (nAChRs) expressed abundantly on BBB capillary endothelial cells, safely triggering receptor‐mediated transcellular transcytosis across the BBB. Once inside the specialized parenchymal matrix, these vesicles utilize their engineered homing ligands to dock onto malignant cells, delivering targeted small interfering RNAs (siRNAs) or small‐molecule inhibitors directly into the tumor core. This engineered precision significantly suppresses tumor growth, minimizes systemic cardiotoxicity and bone marrow suppression, and dramatically extends animal survival compared with free drug administration, establishing a robust blueprint for future translation [159, 160, 161, 162].

#### 5.2.2 The Engineering Frontier: From Natural Exosomes to Synthetic Biomimetic Platforms

While engineered natural exosomes (such as mesenchymal stem cell‐derived or dendritic cell‐derived vesicles) modified with homing peptides have demonstrated remarkable therapeutic efficacy in preclinical models, their transition from laboratory benches to industrial‐scale clinical manufacturing faces severe logistical and regulatory bottlenecks. Primarily, natural exosome isolation suffers from extremely low volumetric yields, complex and unscalable purification protocols (such as density‐gradient ultracentrifugation), and substantial batch‐to‐batch compositional heterogeneity. Furthermore, because natural exosomes carry a highly intricate, poorly defined cocktail of endogenous donor‐cell proteins and noncoding RNAs, their systemic administration introduces serious regulatory risks regarding long‐term immunogenicity and unintended oncogenic priming [163].

To circumvent these translational limitations, the field of cancer nanomedicine has pioneered a next‐generation paradigm shift: the transition from natural exosomes to synthetic biomimetic nanovesicles and hybrid cell‐membrane camouflaged platforms. This engineering evolution aims to marry the robust, scalable, and controllable manufacturing advantages of traditional synthetic liposomes with the superior biocompatibility, long circulating half‐life, and innate organotropic homing capabilities of natural extracellular vesicles (Figure 5B) [164]. Technologically, these advanced platforms are engineered through two primary synthetic methodologies:

##### 5.2.2.1 Top‐Down Cell‐Derived Nanovesicles (CDNs)

Instead of waiting for donor cells to passively secrete rare vesicles, parental cells (such as neutrophils, macrophages, or red blood cells) are subjected to high‐shear mechanical extrusion through sequential polycarbonate membranes or microfluidic channels. This destructive yet controlled processing forces the parental cell membranes to self‐assemble into exosome‐like nanovesicles. CDNs not only match the size and morphology of natural exosomes but achieve a 100‐ to 1000‐fold increase in nanoparticle yield and protein productivity, yielding a highly scalable, economically viable platform for clinical mass production [165, 166, 167].

##### 5.2.2.2 Bottom‐Up Hybrid Biomimetic Liposomes:

Chemists synthesize structurally defined, clinical‐grade lipid bilayers and subsequently fuse them with purified, genetically engineered membrane proteins (such as CD47 antiphagocytic signals or tumor‐targeting integrins) or native cell membranes extracted from leukocytes or patient‐specific primary tumor cells. This fusion creates a highly controlled, hybrid nanovesicle [168, 169].

The clinical superiority of these synthetic biomimetic platforms over natural exosomes is profound. Because the core architecture is synthetic, bio‐engineers can achieve absolute precision in therapeutic cargo loading, packing the vesicular lumen with uniform, high‐density concentrations of chemotherapeutic agents, synthetic siRNAs, or CRISPR‐Cas9 ribonucleoprotein complexes without interference from endogenous cellular debris [170, 171]. Concurrently, by decorating the synthetic lipid bilayer with multifunctional surface ligands, such as the T7 peptide or transferrin alongside tumor‐stroma targeting aptamers, these biomimetic vehicles can systematically cross formidable tissue barriers and selectively release their cytolytic payloads exclusively within the acidic, hypoxic tumor matrix [172]. By resolving the long‐standing conflicts between scalability, safety, and targeting precision, the engineering of synthetic biomimetic nanovesicles represents the defining future of exosome‐based cancer therapeutics, paving a realistic regulatory path toward standardized, off‐the‐shelf precision oncological medicine.

### 5.3 Targeting the Exosomal Secretory and Clearance Pathways as an Oncological Strategy

While exploiting engineered or synthetic biomimetic nanovesicles as precision drug delivery vehicles represents a powerful offensive arm in cancer nanomedicine, a comprehensive therapeutic paradigm requires an equally robust defensive strategy: systematically disrupting the endogenous exosomal networks that tumors utilize to orchestrate local stromal corruption and systemic host wasting (Figure 5C). Given that tumor‐derived vesicles drive angiogenesis, metabolic remodeling, and immune evasion, blocking their biogenesis, intercepting their circulation, or accelerating their systemic clearance has emerged as a cutting‐edge frontier in oncology. This therapeutic approach focuses on neutralizing the malignant secretome rather than using it as a carrier [173].

#### 5.3.1 Pharmacological Inhibition of Vesicular Biogenesis and Secretion

The first line of intervention targets the intracellular machinery responsible for exosome assembly and release. Pharmacological screening has identified several small‐molecule inhibitors that selectively disrupt either the lipid‐dependent or protein‐dependent secretory pathways within malignant cells. Mechanistically, targeting the endosomal neutral sphingomyelinase 2 (nSMase2) pathway, which regulates the synthesis of ceramide required for membrane invagination into multivesicular bodies (MVBs), using the chemical inhibitor GW4869 significantly decreases the absolute secretion of oncogenic sEVs across multiple solid tumors [174, 175].

In parallel, protein‐sorting pathways governed by the Ras‐associated Rab GTPase family can be targeted. Inhibitors such as Manumycin A (a farnesyltransferase inhibitor) suppress the functional activation of Rab27a and Rab27b, effectively halting the docking and fusion of late endosomes with the cancer cell plasma membrane and capturing malignant vesicles within the intracellular matrix [176, 177]. By restricting the molecular shedding of pro‐angiogenic noncoding RNAs and immunosuppressive surface proteins (e.g., PD‐L1), these biogenesis inhibitors successfully sensitize refractory tumors to conventional chemotherapies and standard immune checkpoint blockades in preclinical settings [178].

#### 5.3.2 Circulatory Interception and Accelerated Systemic Clearance

To complement intracellular biogenesis blockade, clinical translation has pioneered methods to physically trap and eliminate tumor exosomes already circulating within the systemic vascular channels. This is primarily achieved through extracorporeal physical interception and monoclonal antibody‐mediated clearance acceleration [179].

In the clinical landscape, advanced medical device engineering has produced the Aethlon ADAPT (adaptive dialysis affinity platform) system, an innovative plasma‐filtration affinity column integrated into continuous venovenous hemofiltration circuits. The matrix of this therapeutic lectin‐affinity column is functionalized with targeted immobilized outer‐membrane lectins or specific antibodies that bind with exceptional affinity to the unique clustered high‐mannose glycans and surface tetraspanins (CD9, CD63) uniquely overrepresented on the lipid bilayers of cancer‐derived exosomes. As the patient's plasma circulates through the device, malignant vesicles are selectively immobilized and retained within the cartridge while healthy blood cells and essential plasma proteins are returned to the systemic circulation, achieving a noninvasive, mechanical “washing” of the patient's circulatory secretome [180]. Concurrently, pharmacology has advanced monoclonal antibody cocktails designed to accelerate the hepatic and splenic clearance of malignant vesicles in vivo. Systemic administration of target‐specific antibodies against exosomal surface antigens, such as Glypican‐1 (GPC1) or anti‐PD‐L1, not only neutralizes their immediate target‐binding capabilities but also decorates the malignant vesicle with active Fc domains [181, 182]. This molecular opsonization triggers robust Fc‐receptor‐mediated phagocytosis by resident Kupffer cells in the liver and red pulp macrophages in the spleen, rapidly purging the circulation of oncogenic cargoes. Furthermore, combining these strategies with therapies that disrupt the CD47‐SIRPα (“don't eat me”) signaling axis, which tumors hijack by expressing high‐density CD47 on their exosome surfaces to evade macrophage surveillance, unleashes a massive engulfment cascade [183, 184]. By transitioning from passive observation to active pharmacological and mechanical eradication of the malignant vesicle pool, targeting the exosomal lifecycle offers an innovative methodology to paralyze tumor communication long before systemic premetastatic niches can form.

### 5.4 Global Landscape of Ongoing Clinical Trials

Driven by both the offensive strategies of utilizing engineered vehicles and the defensive paradigms of targeting endogenous secretory networks, the translational maturation of vesicle technologies has catalyzed a substantial expansion of international, registered human trials. Rather than being confined to narrow diagnostic assays, the contemporary clinical registry encompasses a diversified therapeutic and interceptive portfolio across both systemic and barrier‐constrained malignancies (Table 2).

**TABLE 2:** Clinical trials involving exosome‐based cancer applications.

NCT Number	Status/Phase	Application type	Disease	Intervention/Platform	Objective	Preliminary findings
NCT04720599	Completed/ongoing clinical validation study	Diagnostic liquid biopsy	Prostate cancer	Urinary exosomal RNA‐based ExoDx assay	Evaluate whether urinary exosomal biomarkers improve risk stratification and reduce unnecessary prostate biopsy	Demonstrated clinical utility for prostate cancer risk assessment and improved patient selection
NCT077755344	Ongoing two‐phase pilot study	Molecular monitoring	High‐grade glioma	CSF‐derived extracellular vesicle profiling	Characterize tumor‐associated vesicle biomarkers	Expected to provide longitudinal monitoring of tumor evolution and treatment‐associated phenotype changes
NCT03608631	Phase I clinical trial	Therapeutic engineered exosome delivery	Metastatic pancreatic ductal adenocarcinoma	KRAS G12D siRNA‐loaded mesenchymal stem cell‐derived exosomes (iExosomes)	Evaluate safety, feasibility, and preliminary antitumor activity of targeted KRAS silencing	Early clinical evaluation established feasibility of systemic exosome‐mediated RNA delivery

*Note*: Data sources—ClinicalTrials.gov.

#### 5.4.1 Liquid Biopsy and Diagnostic Registries

Diagnostic trials leveraging natural circulatory or proximal biofluid vesicles heavily dominate the active registries due to their noninvasive nature and minimal regulatory hurdles. For example, large‐scale multicenter trials such as NCT04720599 have successfully validated the clinical utility of urinary exosomal gene expression profiling (e.g., the ExoDx Prostate test) to risk‐stratify prostate cancer patients and eliminate unnecessary biopsies [185]. Similarly, in the context of tissue‐insulated malignancies, trials like NCT07775534 are longitudinally harvesting cerebrospinal fluid (CSF) vesicles from patients with high‐grade gliomas to track target mutations (such as *IDH1* and *EGFRvIII*) and predict spontaneous proneural‐to‐mesenchymal phenotype switching under therapeutic pressure. These trials collectively demonstrate that vesicular multifluid profiling provides an unadulterated circulating proxy for dynamic tumor clonal evolution.

#### 5.4.2 Therapeutic Interventions and Bio‐Engineering Trials

Conversely, therapeutic trials utilizing highly engineered or synthetic biomimetic vesicles are rapidly advancing through early‐phase human testing, establishing clinical safety profiles for targeted oncology. A defining milestone in the offensive drug‐delivery arm is NCT03608631, a Phase I trial evaluating the therapeutic efficacy of iExosomes (mesenchymal stem cell‐derived exosomes engineered with siRNA targeting the *KRAS G12D* mutation) in patients with metastatic PDAC [186]. This trial represents the first regulatory validation of using surface‐chaperoned vesicle platforms to deliver gene‐silencing therapeutics into highly desmoplastic, therapy‐resistant systemic tumor matrices. Simultaneously, the defensive therapeutic strategy, focused on neutralizing rather than exploiting the malignant secretome, is successfully transitioning into clinical settings. The deployment of the Aethlon ADAPT (adaptive dialysis affinity platform) system utilizes specialized extracorporeal lectin‐affinity cartridges integrated into continuous venovenous circuits. These devices physically capture and eliminate circulating tumor‐derived vesicles displaying high‐mannose glycans and PD‐L1 from the patient's plasma, mechanically purging the vascular channels to rescue host adaptive immunity from systemic vesicle‐induced exhaustion. This dual expansion across diagnostic, drug‐delivery, and interceptive clinical pipelines indicates that the conceptual barriers surrounding vesicle translation are being systematically dismantled.

## 6 Unresolved Bottlenecks and Future Roadmap

Despite the profound clinical momentum detailed across the international registry, transitioning the biology of exosome communication from an elegant preclinical narrative into an unassailable clinical reality requires the scientific community to ruthlessly confront three foundational, tech‐limiting bottlenecks that currently clog the translation pipeline. Over the next decade, the paradigm shift from empirical observation to standardized, off‐the‐shelf precision oncology must actively navigate the following structural frontiers:

### 6.1 The Resolution Gap: Transitioning from Bulk Averages to Single‐EV Heterogeneity

Currently, most published multi‐omic datasets, whether profiling exosomal miRNAs for pan‐cancer screening or liquid subtyping via biofluid compartments, rely on “bulk isolation” methods (such as standard ultracentrifugation or precipitation kits). These approaches aggregate billions of vesicles into a single pooled sample, effectively generating a blurred, population‐wide average. However, it is now established that a single cancer cell simultaneously dispatches an intricately heterogeneous armada of vesicles, ranging from classical subpopulations to nonmembranous exomeres and supermeres [187]. Bulk extraction fundamentally obliterates this high‐resolution spatial identity, masking the ultrarare, highly aggressive “driver vesicles” responsible for initiating localized cellular remodeling. Overcoming this roadblock demands the industrial standardization of next‐generation single‐EV analysis (SEA) platforms. Leveraging high‐throughput optofluidic nanoplasmonic imaging, nanofluidic microchips, and single‐vesicle flow cytometry will allow future clinicians to profile the multi‐omic surface marker and transcriptomic cargo configuration of an individual nanoparticle with single‐molecule sensitivity, unlocking an unadulterated resolution of tumor clonal evolution (Figure 6) [188, 189].

**FIGURE 6:**
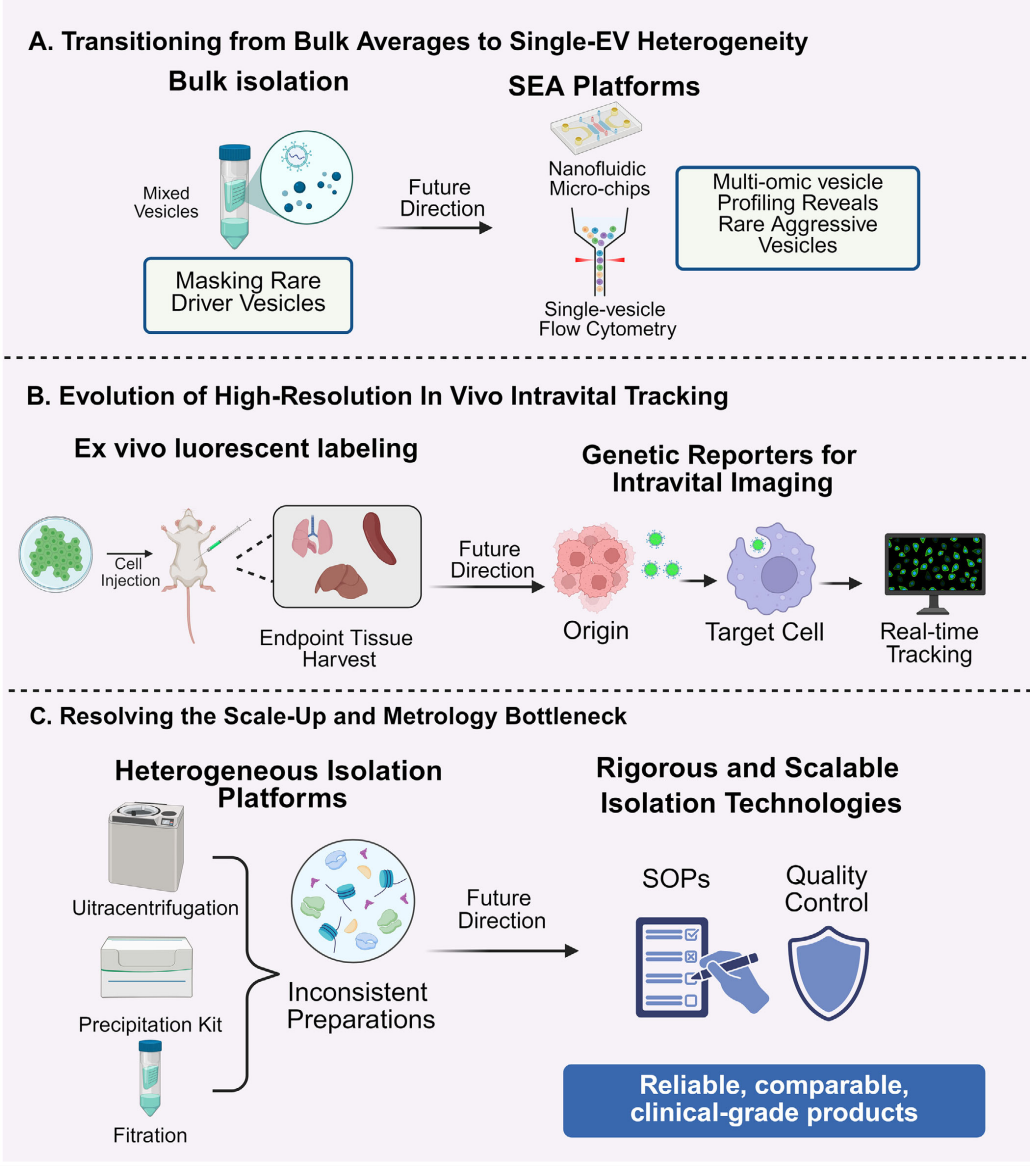
Unresolved bottlenecks and future roadmap for exosome‐based oncology translation. (A) Transitioning from bulk averages to single‐EV heterogeneity. Bulk isolation masks rare functional vesicle populations, whereas single‐EV analysis platforms may enable high‐resolution multi‐omic profiling of individual tumor‐derived vesicles. (B) Evolution of high‐resolution in vivo intravital tracking. Conventional ex vivo labeling and endpoint tissue analysis should be replaced by genetic reporter‐based intravital imaging for real‐time tracking of native exosome origin, biodistribution, and target‐cell uptake. (C) Resolving the scale‐up and metrology bottleneck. Standardized, scalable isolation technologies, rigorous SOPs, and quality‐control benchmarks are required to generate reliable, comparable, clinical‐grade exosome products. *Source*: Created with BioRender.com.

### 6.2 The Spatiotemporal Blindspot: Evolution of High‐Resolution In Vivo Intravital Tracking

A second deep‐seated limitation resides in the structural disconnect between static exosome isolation and dynamic, in vivo spatiotemporal clearance kinetics. The current literature remains heavily saturated with static preclinical methodologies, where exosomes are fluorescently labeled (e.g., with PKH67 or DiO) ex vivo, intravenously injected into mouse models, and analyzed via end‐point tissue harvesting [190]. This crude methodology alters the native vesicular membrane charge, leading to artificial accumulation in the hepatic and splenic reticuloendothelial sanctuaries and obscuring the authentic physiological biodistribution of endogenous tumor vesicles. The field urgently requires the development of noninvasive, high‐contrast, genetically encoded intravital multiplexed imaging modalities. Utilizing advanced bioluminescent reporters (such as split‐luciferase or multiplexed CRISPR‐engineered color‐switching tags) under the control of tumor‐specific promoters will enable the continuous, real‐time tracking of native exosomes from their exact site of biogenesis, through the systemic circulation or across strict endothelial barriers, to their precise single‐cell target integration site in living organisms (Figure 6B) [191].

### 6.3 The Standardization Chasm: Resolving the Scale‐Up and Metrology Bottleneck

Finally, from an industrial and pharmaceutical standpoint, the translation of both natural engineered exosomes and synthetic biomimetic hybrids remains heavily constrained by the lack of globally harmonized standard operating procedures (SOPs) and international metrological benchmarks. Currently, differences in isolation platforms yield radically divergent products contaminated with varying degrees of nonvesicular protein complexes (such as Tamm–Horsfall proteins or albumin) and lipoprotein particles (chylomicrons and VLDLs). This contamination severely compromises reproducibility across clinical trial sites. Bridging this standardization chasm requires the development of rigorous, scalable isolation technologies capable of producing clinical‐grade, cGMP‐compliant vesicles under strict quality‐control metrics. Furthermore, the field must establish standardized molecular reference standards (similar to those utilized in viral vector manufacturing) to evaluate therapeutic potency, absolute particle counting, and vesicle‐to‐cargo loading ratios (Figure 6C) [192, 193].

## 7 Conclusion and Future Perspectives

The evolution of exosome biology from early descriptions of metabolic waste disposal to the contemporary understanding of a highly organized, multidimensional extracellular network marks a paradigm shift in modern oncology. As synthesized throughout this review, the tumor secretome, now recognized as a diverse continuum spanning classical endocytic exosomes and newly identified nonmembranous extracellular nanoparticles such as exomeres and supermeres, serves as a primary engine driving the pan‐cancer macroevolution. By orchestrating a horizontal transfer of multi‐omic regulatory components, these circulating catalysts systematically subvert tissue‐specific physiological boundaries, enforce local stromal rewiring, and fertilize distant premetastatic soils.

However, as the field transitions into the next decade, the clinical translation of these biological insights stands at a critical crossroads. The offensive exploitation of engineered or synthetic biomimetic nanovesicles for targeted, barrier‐crossing drug delivery, paired with defensive interventions aimed at pharmacological biogenesis blockade and extracorporeal affinity clearance, holds immense therapeutic promise. Yet, the realization of this promise remains heavily constrained by structural bottlenecks. Bridging the current “standardization chasm” and overcoming the “resolution gap” through single‐EV analysis and high‐fidelity intravital tracking represent the defining challenges of contemporary nanomedicine.

Ultimately, the future of exosome‐guided precision oncology hinges upon our capacity to reconcile universal vesicular traits with organ‐specific biological constraints. Moving forward, researchers and clinicians strive to transcend bulk‐population averages and static observations, shifting toward a dynamic, single‐molecule resolution of the malignant secretome. By conquering these technological and metrological frontiers, exosome‐targeted strategies will undoubtedly transition from elegant preclinical narratives into standardized, off‐the‐shelf clinical realities, fundamentally redrawing the landscape of cancer diagnosis and therapeutics.

## Author Contributions

L.Z. conceived the topic and provided overall guidance and critical revisions throughout the writing process. J.W., L.W., and K.Y. conducted the literature review, organized relevant studies, and drafted the manuscript. K.Y. prepared the figures. K.X., T.L., T.H., D.W., and T.S. provided valuable comments on the manuscript and contributed to reviewing and editing. All authors have read and approved the final manuscript.

## Funding

This work was supported by the Natural Science Distinguished Young Scholars Fund (Hunan 2024JJ2091, Jiangxi 20242BAB23073); National Clinical Research Fund of Geriatric Disorders (2023LNJJ19); Ganpo Innovative Leading Fund (Medical and Health Sciences, gpyc20250218).

## Ethics Statement

The authors have nothing to report.

## Conflicts of Interest

The authors declare no conflicts of interest.

## Data Availability

The authors have nothing to report.
